# Colorectal cancer drug target prediction using ontology-based inference and network analysis

**DOI:** 10.1093/database/bav015

**Published:** 2015-03-26

**Authors:** Cui Tao, Jingchun Sun, W. Jim Zheng, Junjie Chen, Hua Xu

**Affiliations:** ^1^Center for Computational Biomedicine, School of Biomedical informatics, University of Texas Health Science Center at Houston, Houston, TX 77030, USA and ^2^Department of Experimental Radiation Oncology, The University of Texas MD Anderson Cancer Center, Houston, TX 77030, USA

## Abstract

Identification of novel drug targets is a critical step in drug development. Many recent studies have produced multiple types of data, which provides an opportunity to mine the relationships among them to predict drug targets. In this study, we present a novel integrative approach that combines ontology reasoning with network-assisted gene ranking to predict new drug targets. We utilized colorectal cancer (CRC) as a proof-of-concept use case to illustrate the approach. Starting from FDA-approved CRC drugs and the relationships among disease, drug, gene, pathway, and SNP in an ontology representing PharmGKB data, we inferred 113 potential CRC drug targets. We further prioritized these genes based on their relationships with CRC disease genes in the context of human protein–protein interaction networks. Thus, among the 113 potential drug targets, 15 were selected as the promising drug targets, including some genes that are supported by previous studies. Among them, EGFR, TOP1 and VEGFA are known targets of FDA-approved drugs. Additionally, CCND1 (cyclin D1), and PTGS2 (prostaglandin-endoperoxide synthase 2) have reported to be relevant to CRC or as potential drug targets based on the literature search. These results indicate that our approach is promising for drug target prediction for CRC treatment, which might be useful for other cancer therapeutics.

## Introduction

Drug discovery is a time-consuming and expensive process, especially for complex diseases. In the last decade, in contrast to traditional phenotypic drug discovery, target-based methods for drug discovery have become more common and effective (1). Additionally, drug repurposing, finding new therapeutic uses for old drugs, is another efficient and effective approach to facilitating drug discovery (2). However, the traditional approaches for drug repurposing still mainly depend on phenotypic drug screening or target-based methods using prior knowledge of mechanisms (3, 4). Since the knowledge related to drug action is distributed among different knowledge domains and different databases, it becomes challenging to design effective strategies for revealing the hidden connections between novel drug targets and repurposed drugs. Recently, computational approaches have become one of the major methods for alleviating this issue through the comprehensive integration of heterogeneous knowledge and data, including genetic and genomic data, pharmaceutical data and pathway data. Therefore, these approaches could accelerate the process of revealing the valuable information underlying these complicated data and lead to the identification of promising drug targets and repurposed drugs (2, 5).

Most computational methods focused on revealing new relationships between drugs and diseases based on different biological perspectives such as pathway profiles (6), drug similarities (7) or gene expression data (5, 8). However, drug-disease relationships are not isolated from other relationships since many factors systematically contribute to the determination of the molecular mechanisms underlying drug action. Therefore, it is important to consider different factors comprehensively and interactively when developing effective medications. Thus, in this study, we utilized the semantic web and biological network technologies to integrate the relationships among drugs, genes, diseases, pathways and SNPs into one system for discovering potential drug targets.

The semantic web technology provides several unique benefits for data integration and knowledge inferences. Representing relevant drug and disease associations using semantic web notations will enable flexible data integration among heterogeneous data sets, which is a well-known challenge in the translational science study community (9). The Web Ontology Language (OWL) is a standard ontology language for the Semantic Web that allows drug relevant knowledge to be represented in a machine-understandable way (an ontology), which enables automatic semantic reasoning for drug repurposing (10). The Resource Description Framework (RDF) is a W3C standard for representing data that allows efficient querying and visualization of relationships between biomedical entities (11). RDF itself can be viewed as a graph that can serve as the foundation of network-based analysis. Network-based approaches to human disease and treatment have multiple potential biological and clinical applications, such as novel drug discoveries (12–14) and identification of novel drug targets (15, 16).

Colorectal cancer (CRC) is one of the most commonly diagnosed cancers. It involves multiple genes or proteins that interact with each other, but in which each gene or protein contributes a small ‘risk’ on its own (17). Previous research suggests that the most effective medications should interact with or have influence on several molecular targets, not just one target (18, 19). Thus, we hypothesized that the combination of ontology-based data representation, semantic-based reasoning and network-based prioritization will facilitate the prediction of novel targets for the development of novel CRC therapy. In this study, we first represented the relationships among drugs, diseases, genes, pathways and SNPs in an OWL ontology. We then specified computer rules to infer potential CRC drug targets. From these inferred targets, we prioritized the most promising drug targets for CRC treatment by integrating the relationships between drug targets and CRC disease genes in the context of a human protein–protein interaction (PPI) network. Three of the results are known targets of FDA-approved drugs used to treat CRC. Additionally, some others have been supported to be related to CRC or as potential drug targets based on literature search results. The results indicate that our combination method of ontology and network analysis is promising for the identification of novel drug targets, which may provide valuable information for development of novel CRC treatment.

## Materials and methods

### Collecting FDA-approved CRC drugs, drug classification and drug targets

We first collected the FDA-approved drugs to treat CRC from the cancer drug list (http://www.cancer.gov/cancertopics/druginfo/alphalist), which was compiled by the National Cancer Institute. We further verified the indication and usage information of these drugs using their FDA label files. To learn more about these drugs, we extracted their classification information from the Anatomical Therapeutic Chemical (ATC) classification from KEGG (Kyoto Encyclopedia of Genes and Genomes) (20). The ATC classification provides information for a given drug regarding which organ or system it acts on and/or its therapeutic and chemical characteristics. The KEGG includes several domains of knowledge, such as diseases, genes, pathway, drugs and genomes. It not only provides detailed information for each entity but also consists of the relationships among these entities within each domain or across different domains. Data and relationships in KEGG were mainly collected from literature (21). Drug targets were extracted from the DrugBank (22) and the Therapeutic Target Database (TTD) (23) (Supplementary Table S1).

### Constructing a CRC drug ontology for inferring potential CRC drug targets

We have created an OWL ontology for drugs, diseases, genes, pathways and SNPs, and modeled the relations among them using data downloaded from the PharmGKB database (May 2013 version) (24). The PharmGKB is a pharmacogenomics knowledge resource, which collects, curates and disseminates knowledge about the impact of human genetic variation on drug responses (25). We used data extracted from the relationship file and pathway file from PharmGKB. The files were first loaded into a relational database. We then manually defined a PharmGKB ontology that contains meta-level classes such as drug, disease, gene, pathway and SNP. Object properties were also defined to describe the relations between the classes (e.g. associatedWithGene, associatedWithDisease, etc.) We then developed a Java converter that reads the data from the relational database and stores the data at the instance level in OWL. Each entity (e.g. a particular gene or disease) is represented as instances (OWL individuals) of the corresponding class. Relationships between these individuals are defined using RDF triples with the defined object property (e.g. GP1BB associatedWithGene COL3A1).

**Figure 1.**
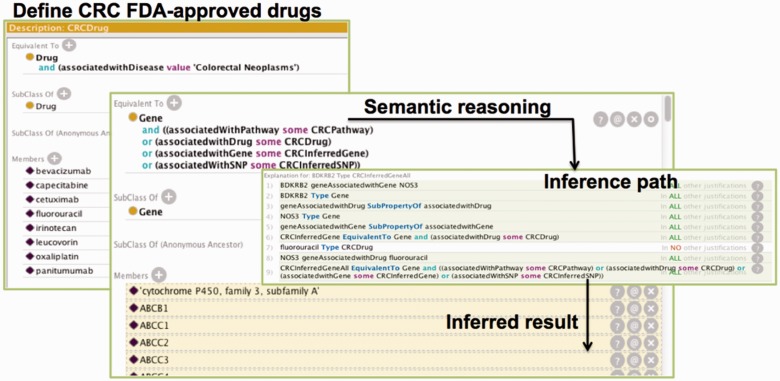
The process of semantic reasoning using CRC as an example. The process involved three steps. We first utilized the OWL definition to define the CRC drugs in Protégé ontology editor. The ‘Equivalent to’ section shows the semantic definition of the CRC Drug class whereas the ‘Members’ section shows a partial list of all the drugs that are members of the class. Then, we employed DL rules to determine the inference path. This figure only shows the overall rule for inferring the possible genes that are relevant to CRC drugs. Additional rules were defined to infer CRC relevant pathways, SNPs and genes in the ontology. Finally, we used Pellet to infer potential CRC target genes. The ‘Equivalent to’ section shows a DL rule for finding the potential CRC target a gene whereas the ‘Members’ section (in yellow background) shows the inferred target genes.

On top of this PharmGKB ontology, we further specified a drug repurposing application ontology for CRC. A new CRC Drug class has been created to serve as the basis of our drug target inference. We defined that a CRC drug is a drug that is associated with Disease Colorectal Neoplasms. In addition, all the FDA approved CRC drugs are listed as instances of this class. We further specified OWL DL (description logic) rules to infer possible CRC drug target genes. A CRC relevant gene must be a gene that associates with either a CRC associated pathway, drug, gene or SNP. More rules are specified in the ontology to automatically locate genes that are relevant to CRC drugs as well as their associated SNPs, pathways, genes and diseases. For example, we can define the SNPs that are directly associated with any CRC drug (we call these CRCSNP) using the DL rule *SNP and associatedWithDrug some CRCDrug*. In addition, we can find the CRCSNPs that are at most two nodes away by using this DL rule:

*SNP and ((associatedwithDrug some CRCDrug) or (associatedwithGene some CRCGene) or (associatedWithSNP some CRCSNP))*, where CRCGene is defined similar as CRCSNP. With these DL rules, we can find genes that directly or indirectly connect with any CRC Drug instance. In this project, we only consider the entities that are at most two nodes away from the CRC drug nodes. Figure 1 summarized the process. The ontology can be accessed from our web site: https://sbmi.uth.edu/ontology/project/drug-repurposing.htm.

### Functional analysis of potential drug targets revealed by CRC ontology search

To assess if the genes that encode the potential CRC drug targets inferred above were enriched in the pathways related to the CRC, we performed the KEGG pathway enrichment analysis using an online tool called WebGestalt (version 2) [29]. The WebGestalt performed the hypergeometric test followed by the Benjamini–Hochberg method to control type I errors [30]. We selected those pathways that have adjusted *P*-values of <0.001 as the enriched pathways. To make the analysis biologically meaningful, we considered only those KEGG pathways containing five or more genes.

### Collecting CRC disease genes

To create a specific and comprehensive list of CRC disease-causing genes (CRC disease genes), we extracted disease genes from three resources: the Cancer Gene Census (CGC) (26), the Online Mendelian Inheritance in Man (OMIM) (27) and the Genetic Association database (GAD) (28). The CGC is an ongoing effort to catalog genes with mutations that have been causally implicated in cancer. We downloaded the list of known cancer genes from the CGC website in December 2013 and extracted 23 genes associated with CRC. The OMIM database was the first database to collect all known diseases with their genetic components. It provides a precise and comprehensive summary of clinical and genetic information on cancer. From its description of CRC, 12 genes were extracted (Downloaded in December 2013). The GAD is a resource of summarized human genetic association studies of complex diseases and disorders. From GAD, we extracted 34 genes at least with one positive association with CRC (downloaded in December 2013). After combining these lists, we finally arrived at 56 genes as CRC disease genes. Only three genes were common to all three data sources (Supplementary Table S2).

### Ranking CRC candidate drug targets

To identify the most promising targets among the ontology-driven CRC potential drug targets, we implemented network neighborhood modeling to prioritize the potential drug targets. More specifically, we utilized the relationships among drug targets and CRC disease genes in the context of the human PPI network. The ranking method was mainly based on the hypothesis that the closer the targets are to causal genes, the more efficiently drugs will act. The human PPI network provides a comprehensive platform to investigate the association between CRC disease genes and drug targets (18).

Here, we first downloaded the human PPIs from the Protein Interaction Network Analysis platform (PINA v2.0) (downloaded in September 2013) (29), which were derived from human-related experiments. After filtering out the PPIs without experimental evidence and removing redundancies and self-interactions, we built a human PPI network that included 101 219 edges and 12 978 proteins. Second, we mapped the ontology-driven candidate drug targets and CRC disease genes onto the human PPI network. Third, we ranked the candidate targets based on the fraction of CRC disease genes in their neighborhood. For example, for a given candidate target, we collected the nodes that have direct links with it as its first-degree neighbors (N) and then counted the number of neighbors belonging to the CRC disease genes (n). Based on the two numbers, we calculate the fraction (n/N) to represent the fraction of disease genes around the drug target. Previous studies suggested that the fraction of disease genes is enriched at the first, second and third shortest path distances in the neighborhood of drug targets. And similarly, the fraction of drug targets is enriched at the first, second and third shortest path distances in the neighborhood of disease genes (18, 30). Therefore, we only utilized their relationships at the first-, second- and third-level to rank these candidate drug targets, respectively. To integrate the three sets of rankings, we employed a robust rank aggregation (RRA) method, which is implemented in the R package called RobustRankAggreg (31). The RRA method can detect genes that are ranked consistently better than expected by chance. The method assigns a *P*-value to each gene based on significant scores, which shows how much better it is positioned in the ranked list than expected by chance designated as potential CRC targets.

## Results

### Overview of the computational framework and related data

**Figure 2.**
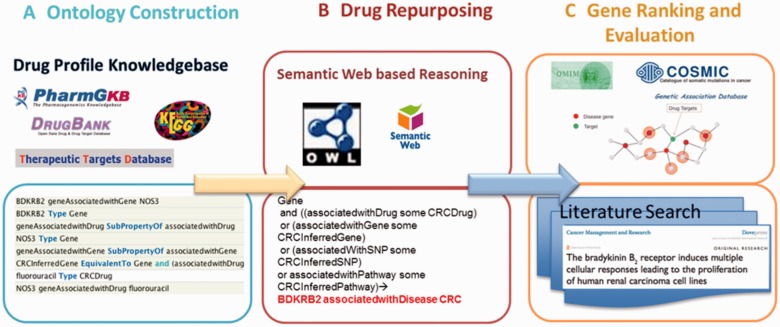
Computational framework for predicting the potential drug targets using CRC as an example. The framework involves three main steps: 1) ontology construction and collection of CRC drugs and their targets, 2) semantic reasoning and 3) network-based gene prioritization.

In this study, we developed a computational framework to integrate complex relationships among different types of data and infer potential drug targets by using semantic web technology, and to improve performance through network neighborhood effect modeling. In this study, we utilize CRC as a proof-of-concept use case to evaluate this approach. Figure 2 illustrates each step in this framework. We first constructed the ontology based on the relationships among drugs, genes, diseases, pathways and SNPs from PharmGKB and collected FDA-approved drugs and their targets from DrugBank. Second, we collected CRC disease genes (Supplementary Table S2) from multiple data sources and inferred the CRC potential drug target genes using semantic reasoning methods. Finally, we further prioritized the inferred genes based on their relationships with CRC disease genes in the context of PPI networks and performed literature searches to provide independent evidence for the top ranked genes.

In this study, considering that the FDA approved drug have well-characterized pharmacology, we employed the FDA-approved drugs for one disease as the input into the ontology system to infer the genes that might have significant association with drug targets, associated SNPs, genes and pathways that reflect their molecular mechanisms. Then we hypothesized that these genes may have potential as drug targets. Therefore, we collected 10 FDA-approved drugs to treat CRC that existed in PharmGKB database, their classifications and their targets (Table 1). Among the 10 drugs, eight belong to antineoplastic agents (ATC class L01), three belong to the monoclonal antibodies (L01XC), two to the antimetabolites (L01B), one to the oxaliplatin (L01XA), one to the protein kinase inhibitors (L01XE) and one to the other antineoplastic agents (L01XX). Of the other two, the drug aflibercept belongs to the antineovascularization agents (ATC class S01LA) and leucovorin calcium belongs to the detoxifying agents for antineoplastic treatment (ATC class V03AF). Among the 10 drugs, nine have 40 unique targets that were collected from two drug-target databases, DrugBank and TTD.

**Table1.** Summary of FDA-approved drugs used to treat CRC and their targets

Drug name	PharmGKB ID	ATC classification	Targets from DrugBank	Targets from TTD	Number of targets
Bevacizumab	PA130232992	L01XC07	C1QA, C1QB, C1QC, C1R, FCGR1A, FCGR2A, FCGR2B, FCGR2C, FCGR3A, FCGR3B, VEGFA		11
Capecitabine	PA448771	L01BC06	TYMS	TYMS	1
Cetuximab	PA10040	L01XC06	C1QA, C1QB, C1QC, C1R, C1S, EGFR, FCGR1A, FCGR2A, FCGR2B, FCGR2C, FCGR3A, FCGR3B	ABCC1, EGFR	13
Fluorouracil	PA128406956	L01BC02	TYMS	DPYD	2
Irinotecan hydrochloride	PA450085	L01XX19	TOP1, TOP1MT, TYMS	TOP1	3
Leucovorin calcium	PA450198	V03AF03	TYMS	TYMS	1
Oxaliplatin	PA131285527	L01XA03			0
Panitumumab	PA162373091	L01XC08	EGFR	EGFR, GLRB, GUCY2C	3
Regorafenib	—	L01XE21	ABL1, BRAF, DDR2, EPHA2, FGFR1, FGFR2, FLT1, FLT4, FRK, KDR, KIT, MAPK11, NTRK1, PDGFRA, PDGFRB, RAF1, RET, TEK		18
Aflibercept	—	S01LA05	PGF, VEGFA, VEGFB	KDR	4

### One hundred and thirteen potential drug targets

Using the ontology-based inference from PharmGKB data, we have inferred 113 potential genes (Supplementary Table S3). To assess whether these 113 ontology-inferred genes were significantly associated with CRC, we compared them with the previously defined 56 CRC disease genes and the 40 target genes encoding the 40 known targets of the 10 FDA-approved CRC drugs. Among the 113 genes, eight were present among the 56 CRC disease genes (*CCND1*, *MSH6*, *IL8*, *KRAS*, *MTHFR*, *MTRR*, *PTGS2* and *TP53*). The 56 CRC disease genes were collected from three disease genetic association databases including CGC, OMIM and GAD. The data in these databases were curated by experts and therefore were more likely to be actual CRC disease genes.

The 113 ontologies-inferred genes were enriched significantly with CRC disease genes when compared with the 20 737 human protein-coding genes, which was more than expected by chance (Hypergeometric test *P*-value: 4.96 × 10^−^^10^). Similarly, among the 113 genes, there were nine genes in common with the genes that encode the 40 CRC drug targets (*ABCC1*, *DPYD*, *EGFR*, *FCGR2A*, *FCGR3A*, *TOP1*, *TYMS*, *VEGFA* and *VEGFB*). Thus, the ontology-inferred genes were also significantly enriched with CRC drug target genes when compared with the 20 737 human protein-coding genes (*P*-value: 7.15 × 10^−^^13^).

To further examine the functional characteristics of these 113 genes, we performed a KEGG pathway enrichment analysis using the tool WebGestalt. We identified a total of 30 pathways that are significantly enriched in these 113 genes (Table 2). These pathways can be grouped into five major biological processes according to the KEGG BRITE pathway hierarchies. Two of the pathways belong to cellular processes, 4 to environmental information processing, 10 to human diseases (nine cancer-related and one infectious disease), 12 to metabolism and 2 to organismal systems. Considering that our ontology knowledge is mainly from PharmGKB, the observation that the majority of these pathways belong to drug metabolism and cancer is not surprising. More interestingly, carbohydrate metabolic-related pathways such as ‘ascorbate and aldarate metabolism’, ‘pentose and glucuronate interconversions’ and ‘starch and sucrose metabolism’ were also observed in these genes, which were consistent with previous studies that reported that the glycemic load and carbohydrate intake are associated with risk of CRC (32). In addition, the ‘bile secretion’ pathway (adjusted *P*-value: 9.85 × 10^−^^15^) was also significantly enriched in these 113 genes. This pathway has been reported to play an important role in the CRC pathogenesis as evidenced by epidemiological and experimental studies (33–35). In addition, the pathway ‘steroid hormone biosynthesis’ (adjusted *P*-value: 1.73 × 10^−^^21^) is also enriched in these 113 genes, which is consistent with the results observed in the colon carcinoma cell lines DLD1 and SW480 after treatment with β-catenin siRNA (36). Those observations highlighted that these 113 potential genes might be involved in the drug action in CRC treatment.

**Table 2.** KEGG pathways enriched significantly in the 113 genes

KEGG pathway^a^	Adjusted *P*-value^b^
Drug metabolism—other enzymes^M^	1.37 × 10^−42^
Metabolic pathways^M^	4.58 × 10^−23^
Metabolism of xenobiotics by cytochrome P450^M^	6.63 × 10^−22^
Steroid hormone biosynthesis^M^	1.73 × 10^−21^
Retinol metabolism^M^	9.48 × 10^−21^
Drug metabolism—cytochrome P450^M^	5.10 × 10^−20^
Bladder cancer^D^	5.21 × 10^−17^
Ascorbate and aldarate metabolism^M^	5.21 × 10^−17^
Pentose and glucuronate interconversions^M^	3.73 × 10^−16^
ErbB signaling pathway^E^	1.73 × 10^−15^
Porphyrin and chlorophyll metabolism^M^	6.00 × 10^−15^
Other types of O-glycan biosynthesis^M^	9.85 × 10^−15^
Bile secretion^O^	9.85 × 10^−15^
Starch and sucrose metabolism^M^	4.35 × 10^−14^
Pancreatic cancer^D^	4.78 × 10^−13^
ABC transporters^E^	5.31 × 10^−13^
Pathways in cancer^D^	7.13 × 10^−12^
Pyrimidine metabolism^M^	9.97 × 10^−12^
Prostate cancer^D^	6.39 × 10^−9^
Non-small cell lung cancer^D^	9.90 × 10^−9^
Glioma^D^	2.94 × 10^−9^
Endometrial cancer^D^	3.63 × 10^−7^
Renal cell carcinoma^D^	1.55 × 10^−6^
Melanoma^D^	1.60 × 10^−6^
Gap junction^C^	4.99 × 10^−6^
Cytokine-cytokine receptor interaction^E^	7.82 × 10^−6^
GnRH signaling pathway^O^	8.13 × 10^−6^
Focal adhesion^C^	1.65 × 10^−5^
Hepatitis C^D^	2.98 × 10^−5^
MAPK signaling pathway^E^	7.00 × 10^−4^

^a^The capital letters beside the pathway names are the abbreviation of the KEGG category names at the first-level. C, cell communication, E, environmental information processing, D, human diseases, M, metabolism, O, organismal systems.

^b^Adjusted *P*-value was corrected from nominal *P*-values by Benjamini–Hochberg multiple testing corrections.

### Fifteen promising drug targets

Starting from the 113 genes, we performed gene ranking based on their neighborhood of CRC disease genes in the context of one human PPI network. To assess the association between the 113 ontology-inferred genes and the CRC, we employed the relationships between them and CRC disease genes. Considering that the majority of drug targets has shortest path lengths ranging from one to three (18, 30), we mainly ranked these 113 genes at the first-, second- and third-degree level, respectively, and then integrated their rankings by a novel RRA method from a R package called RobustRankAggreg (31).

Among the 113 genes, 15 genes had significant *P*-values (Table 3). Among them, three encode known CRC drug targets including EGFR, TOP1 and VEGFA. The EGFR is the target of the drugs cetuximab and panitumumab; TOP1 is the target of the drug irinotecan and VEGFA is the target of the drugs aflibercept and bevacizumab. Besides, the CCND1 (cyclin D1) is the target of the drug arsenic trioxide, which is used to treat leukemia. The PTGS2 (prostaglandin-endoperoxide synthase 2) is the target of multiple drugs such as lenalidomide, pomalidomide and thalidomide. The lenalidomide is used for treating lymphoma. Both pomalidomide and thalidomide are used for treating multiple myelomas and other plasma cell neoplasms. The *CCND1* is a well-recognized oncogene that is amplified and/or overexpressed in a substantial proportion of human cancers including colon, prostate and breast (37). Therefore, it might be a promising anti-cancer therapeutic target (38). The gene PTGS2 encodes prostaglandin G/H synthase-2, which catalyzes the first two steps in the metabolism of arachadonic acid. It is overexpressed in many types of cancer such as colon, stomach, breast and lung (39). Additionally, PTGS2 has three variations with pharmacogenomics significance (rs20417, rs5275 and rs689466) (40). Therefore, inhibiting it with drugs such as aspirin, celecoxib and ibuprofen might have potential for the prevention and treatment of cancer (41).

**Table 3.** Genes encoding the 15 promising drug targets

Rank	Gene Symbol	*P*-value^a^
1	*TP53*	5.06 × 10^−6^
2	*EGFR*	1.37 × 10^−4^
3	*UBE2I*	1.37 × 10^−4^
4	*CCND1*	6.33 × 10^−4^
5	*TOP1*	6.33 × 10^−4^
6	*MECP2*	5.06 × 10^−3^
7	*IMPDH2*	6.74 × 10^−3^
8	*NOS3*	6.74 × 10^−3^
9	*XRCC1*	6.74 × 10^−3^
10	*GNAS*	0.0087
11	*VEGFA*	0.0139
12	*PTGS2*	0.0207
13	*CFH*	0.0296
14	*MSH6*	0.0437
15	*GSTP1*	0.0469

^a^*P*-value was calculated based on score distribution.

## Discussion

In this study, we introduce a computational framework to integrate ontology-based data representation, semantic reasoning and network-based gene prioritization for predicting potential drug targets. In the this article, we represented data from the PharmGKB data tables using an OWL ontology. To illustrate that the framework is implementable, we utilized CRC as an example. Starting from FDA-approved CRC drugs and the relationships among drugs, genes, diseases, SNPs and pathways from PharmGKB, the system inferred 113 genes that could be relevant to CRC based on a set of DL rules defined in the ontology. We then further integrated these genes in the context of PPIs, and further inferred 15 potential drug targets for CRC. Some of them are known targets of FDA-approved drugs; others have been reported to be relevant in CRC or CRC treatment. These results demonstrate that the computational framework effectively integrates various types of data and different technologies to predict potential drug targets. In this novel framework, we combined the ontology-based reasoning and network-based prioritization to predict potential drug targets, which can be applied to other diseases as well.

Though the framework effectively predicted potential drug targets, there are ways to improve this approach. First, integrating more relationships among drugs, genes, diseases, SNPs and pathways from more relevant data sources such as DrugBank and KEGG into the ontology system will provide more choices for determination of inference paths during semantic reasoning. In addition, we can take the types of relationships among drugs, genes, diseases, SNPs and pathways into consideration. Our current system treats all types of relations equally. The inferred results could be improved if different types of relations are considered differently. Second, the accuracy and complement of disease genes and drug targets plays a critical role in the network-based prioritization. In the future, we will expand the disease gene source to include for example the Catalogue Of the Somatic Mutations in Cancer (42) and the Comparative Toxicogenomics Database (43) and manually check relevant publications to obtain the most promising disease genes. For the drug targets, we will manually check FDA drug labels and relevant publications to determine the proteins that are responsible for the desired pharmacological effects. Finally, in this framework, we utilized the PPIs from the PINA database that include physical associations, genetic associations and enzymatic reactions. In the future, we will further test if disease genes and targets are preferentially found in particular associations. Besides improving performance, we will expand the prediction of drug targets to drug repurposing, and finally pursue clinical trials for several promising drugs after critical assessment for these repurposed drugs.

## Conclusion

In this article, we present our work on using ontology and network analysis methods to infer potential CRC-relevant genes. We inferred 113 potential CRC drug targets, of which 15 were selected as promising drug targets based on network-assisted ranking, including some genes that are supported by previous studies. The result indicates that our approach is promising for drug target prediction for CRC treatment.

## Supplementary Data

Supplementary data are available at *Database* Online.
